# A study on Chinese college students’ acceptance of brain-computer interface technology and its underlying logic—an empirical analysis based on the extended UTAUT model

**DOI:** 10.3389/fnhum.2026.1884532

**Published:** 2026-08-21

**Authors:** Jing Gao, Jianfeng Hu, Yang Chai, Weiyong Ning

**Affiliations:** Yantai Institute of Science and Technology, Yantai, China

**Keywords:** brain-computer interface, higher education, mixed methods, neuroethics, technology acceptance, UTAUT model

## Abstract

**Objective:**

To examine the acceptance and influencing factors of brain-computer interface (BCI) technology among Chinese college students based on the extended Unified Theory of Acceptance and Use of Technology (UTAUT) model.

**Methods:**

A questionnaire survey was administered to 800 students recruited from 10 universities across eastern, central, and western China using a convergent mixed-methods design. Additionally, 40 students were selected for semi-structured in-depth interviews. We analyzed the quantitative data using partial least squares structural equation modeling (PLS-SEM) and the qualitative data using thematic analysis.

**Results:**

Teacher support (*β* = 0.337, *p* < 0.001), personal innovativeness (*β* = 0.219, *p* < 0.001), and effort expectancy (*β* = 0.156, *p* < 0.001) were positively associated with behavioral intention, while social influence showed a smaller, marginally significant association (*β* = 0.121, *p* = 0.049). Neither performance expectancy nor facilitating conditions turned out to be significant predictors, and neuroethical concerns did not reach significance either (*β* = −0.044). The model still accounted for a substantial 62.6% of the variance in behavioral intention (R^2^ = 0.626). On the qualitative side, four broad barrier categories emerged from the data: privacy anxiety, technological unfamiliarity, insufficient institutional readiness, and the absence of ethical review.

**Conclusion:**

The findings reveal that BCI technology acceptance follows a distinct psychological mechanism from traditional IT. Teacher support, rather than performance expectancy, emerges as the strongest predictor. Four main barriers were identified: privacy anxiety, technological unfamiliarity, insufficient institutional readiness, and the absence of ethical review. The study expands the UTAUT framework for neurotechnology applications in higher education and provides evidence-based recommendations for implementation.

## 1 Introduction

The brain-computer interface (BCI) technology provides a direct communication pathway between the human brain and external devices. It bypasses the conventional peripheral nerve and muscle output pathways, thus enabling the information interaction between brain and computer systems. There are three main applications of BCI technology in education: monitoring of cognitive states, neurofeedback training, and adaptive learning systems (Wolpaw and Wolpaw, 2012; Portillo-Lara et al., 2021).

Using non-invasive EEG, we can track students’ attention levels, cognitive load, and engagement in real time, which gives us objective data to support individualized teaching. Building on that, neurofeedback training—rooted in operant conditioning—lets students learn to regulate their own EEG activity, and this has been shown to improve attention quality, working memory capacity, and cognitive flexibility (Escolano et al., 2014; Zoefel et al., 2011). When you then combine real-time BCI monitoring with AI algorithms, the platform becomes something like an intelligent tutor, one that adapts both the learning content and the pace on the fly in response to each student’s cognitive state.

With more than 47 million students enrolled, China’s higher education system is the largest in the world. At that scale, the opportunities that brain-computer interface (BCI) technology opens up for teaching are enormous. The China Education Modernization 2035 plan creates supportive policy frameworks for new educational technologies, which calls for accelerating the convergence of educational informatization and AI. However, the key to the success of BCI in higher education institutions is the willingness of faculty and students to adopt and use it.

A seminal conceptual model in the area of technology acceptance research is the Unified Theory of Acceptance and Use of Technology (UTAUT) that was proposed by Venkatesh et al. (2003). The model is based on eight major theories such as technology acceptance model (TAM), theory of planned behavior, motivation model and theory of diffusion of innovation, forming an integrated explanatory framework. Venkatesh et al. (2012) subsequently extended UTAUT to UTAUT2 by adding hedonic motivation, price value, and habit for consumer technology contexts. More recently, Dwivedi et al. (2019) and Venkatesh (2022) have called for further refinements to UTAUT to address emerging technologies such as artificial intelligence and neurotechnology, leading to what Venkatesh (2022) refers to as the unified theory of acceptance, use, and exploitation (UTAUTX). In this study, we build upon the foundational UTAUT framework while integrating three additional constructs specifically tailored to the BCI context.

In authentic education contexts, UTAUT and its expanded versions are extensively validated. According to Tian et al. (2024), Chinese graduate students’ acceptance of AI chatbots was most strongly predicted by performance expectancy (*β* = 0.44). Chao and Chen (2023) discovered comparable outcomes for nursing students’ mobile learning. After systematically reviewing the literature of UTAUT, Tamilmani et al. (2021) confirmed the consistency of the predictive power of its core constructs across diverse educational technology contexts. The latest study by Klimova and Pikhart (2025) in Frontiers in Psychology explored how AI affects students’ well-being, expanding the boundaries of UTAUT application in education.

BCI technology is widely applied in medical rehabilitation. However, the study of its acceptance within education is just beginning. BCI interacts directly with the brain and raises neuroethical issues which traditional technology acceptance models have not fully addressed, such as privacy, autonomy, dignity, identity, and data security (Ienca and Haselager, 2016; Klein, 2015; Glannon, 2014). In addition, most of the studies are conducted in Western countries, ignoring the different educational systems and cultures in developing countries such as China. Faculty acceptance of BCI also has a large impact on the implementation of BCI in education.

In light of the above background, the following research questions are posed:

1 What are the key determinants influencing the acceptance of BCI technology among Chinese college students?
2 How do neuroethical concerns shape the intention to adopt this technology?
3 What differences exist across different groups of students?
4 How can root cause analysis inform the development of actionable implementation strategies?

This study makes three contributions. First, it introduces neuroethical concerns as a distinct construct in the UTAUT model to address issues specific to neurotechnology. Second, it explores acceptance patterns among students of different educational levels and backgrounds. Third, it introduces root cause analysis to identify obstacles and provides new tools for technology acceptance research.

The remainder of this paper proceeds as follows. Section 2 presents the research model and hypotheses. Section 3 details the research methods. Quantitative and qualitative findings are reported in Section 4. Section 5 discusses these findings, and Section 6 concludes the paper and highlights practical implications.

## 2 Research model and hypotheses

### 2.1 Theoretical basis

Since Venkatesh et al. (2003) first proposed UTAUT, it has been applied extensively in technology acceptance research across a wide range of fields—new media, healthcare, business, and education among them. The original framework highlights four core determinants of behavioral intention and actual use: performance expectancy, effort expectancy, social influence, and facilitating conditions. By synthesizing eight major technology acceptance theories, UTAUT forms a unified model with strong explanatory power.

Venkatesh et al. (2012) extended UTAUT by three more variables, which are hedonic motivation, price value and habit. The additions are aimed specifically at consumer acceptance of technology. However, the original four core UTAUT constructs are still closely associated in learning environments. The evolving landscape of UTAUT2 over the past decade was systematically reviewed by Tamilmani et al. (2021), confirming the framework’s continuous adaptability within educational technology adoption studies. More recent developments have seen scholars propose UTAUT refinements, incorporating additional moderators and boundary conditions to address the unique characteristics of emerging technologies (Venkatesh, 2022). Furthermore, Tamilmani et al. (2021) systematically reviewed consumer technology acceptance research using UTAUT2, while Dwivedi et al. (2020) developed a meta-UTAUT framework to address emerging technologies such as artificial intelligence and neurotechnology. More recently, Salam et al. (2026b) proposed the Unified MOOC Utilization Model (UMUM), which integrates DeLone and McLean’s IS success model with task-technology fit constructs to explain technology adoption in higher education contexts. The UMUM framework highlights the importance of aligning technological affordances with pedagogical requirements—a principle directly applicable to BCI deployment in educational settings. These evolving frameworks underscore the need for context-specific adaptations of technology acceptance theory. In a related extension, Farooq et al. (2017) proposed the UTAUT3 model by integrating personal innovativeness into the UTAUT2 framework.

As a nascent interdisciplinary discipline, neuroethics specializes in exploring the ethical issues caused by neuroscience and technology. Ienca and Haselager (2016) point out that due to the unique character of neural data, existing privacy frameworks are completely inadequate. Unlike traditional personal data, neural information may disclose an individual’s purposeful direction, psychological condition, and mental content, creating previously unheard-of privacy issues. Klein (2015) presents the idea of “internal coercion” to characterize circumstances in which technology affects decision-making processes in a way that undermines genuine choices.

Glannon (2014) summarizes ethical issues related to BCI technology into four categories: medical safety, privacy and security, personal identity, and agency and responsibility. Haselager et al. (2009) proposed, long before the large-scale application of BCI technology, the basic framework of BCI ethical analysis, pointing out that the challenges of neurotechnology to autonomy, privacy, and identity are different from any previous technology. Lavazza and Giorgi (2023) provide a philosophical foundation for the right to mental integrity in the age of neurotechnologies, arguing that any unauthorized access to neural data constitutes a violation of the fundamental right to cognitive liberty.

### 2.2 BCI technology vs. conventional educational technologies

Before formulating our hypotheses, it is essential to articulate how BCI technology fundamentally differs from conventional educational technologies and why traditional adoption mechanisms may not fully apply. We identify five key distinctions:

1 Direct brain-device interface: Unlike traditional technologies that operate through peripheral input devices (keyboard, mouse, touchscreen), BCI establishes a direct neural pathway. This eliminates familiar interaction paradigms and creates novel cognitive demands that users cannot easily extrapolate from prior experience.
2 Unprecedented ethical stakes: BCI access to neural data raises concerns about mental privacy, cognitive liberty, and identity integrity that no previous educational technology has encountered. These neuroethical dimensions introduce a qualitatively different barrier category.
3 Lack of tangible performance cues: While students can readily envision how a learning management system or mobile app improves their studies, BCI’s educational benefits remain abstract and futuristic. Performance expectancy cannot operate as a strong driver when the performance link is speculative.
4 High perceived risk and uncertainty: BCI involves physical devices interacting with the human body, activating risk perceptions that software-only technologies do not trigger. This bodily dimension elevates trust requirements.
5 Nascent institutional infrastructure: Unlike established technologies supported by campus IT services, BCI lacks standardized training, support protocols, and governance frameworks, making institutional readiness a particularly salient concern.

These distinctions suggest that BCI adoption involves a fundamentally different decision calculus than conventional educational technology adoption, justifying the need for an extended theoretical framework with constructs that capture these unique dimensions.

### 2.3 Research model

Based on the above literature review, this study suggests an extended UTAUT model that adds three constructs: neuroethical concerns, personal innovativeness, and teacher support to the traditional four core constructs. Below we articulate the theoretical rationale for each construct’s inclusion.

Performance Expectancy (PE) refers to the degree to which users believe that using the system will improve their performance. Existing studies have consistently shown that performance expectancy is the strongest predictor of technology acceptance intention (Venkatesh et al., 2003; Tamilmani et al., 2021; Dwivedi et al., 2019). In educational contexts, students evaluate whether BCI technology can improve their learning and academic performance. However, as argued in Section 2.2, BCI’s abstract and nascent nature may weaken this predictive pathway.

Effort Expectancy (EE) refers to the system’s ease of use. This construct reflects how users perceive the amount of mental work necessary to become proficient with a new technology.

Social influence (SI) measures the degree to which a user believes social influencers think they should utilize the system. In collectivist cultures, social influence can carry greater weight.

Facilitating conditions (FC) refer to the organizational and technical support available to users. In educational settings, this covers institutional resources, technical assistance, and relevant policy mechanisms.

Personal innovativeness (PI) captures an individual’s willingness to try out and adopt new technologies. Drawing on the foundational work of Agarwal and Prasad (1998) and the UTAUT3 extension proposed by Farooq et al. (2017)—which explicitly incorporated PI as an eighth core predictor alongside the UTAUT2 constructs—this variable reflects a person’s general openness to technological change. Farooq et al. (2017) demonstrated that PI significantly enhances the explanatory power of the UTAUT framework, accounting for approximately 66% of variance in technology acceptance. We include PI because BCI represents a radical innovation far beyond incremental technological improvements. Rogers' (2003) diffusion of innovation theory posits that personal innovativeness is especially critical for early-stage technologies with high uncertainty. For BCI—a technology most students have never encountered—willingness to experiment becomes a decisive adoption factor. Meta-analytic evidence confirms that PI has stronger effects for emerging technologies than for mature ones (Blut et al., 2022).

Neuroethical concerns (NC) form a multidimensional construct that addresses the distinct ethical challenges raised by neurotechnology—privacy, autonomy, identity, data security, informed consent, and equity among them. We developed this construct specifically for the present study, informed by the ethical systematic review framework for BCI proposed by Burwell et al. (2017). Unlike general technology anxiety or privacy concerns, neuroethical worries are uniquely grounded in the brain’s status as the locus of personal identity and cognitive autonomy (Ienca and Haselager, 2016). The inclusion of NC directly responds to calls for ethics-integrated technology acceptance models in the neurotechnology domain (Goering et al., 2021; Ligthart et al., 2023).

Teacher support (TS) reflects the encouragement and practical help that students feel they receive from their instructors when adopting BCI technology. We incorporate TS because, unlike self-directed consumer technology adoption, educational technology adoption is fundamentally shaped by teacher endorsement (Habibi et al., 2023). This emphasis on instructor-mediated adoption aligns with the Integrated Collaborative Reflection Model (ICRM) proposed by Salam et al. (2026a), which posits that collaborative reflection—supported by teacher guidance and institutional scaffolding—is a core mechanism through which students develop readiness to engage with novel educational technologies. In the Chinese educational context, where teacher authority carries significant cultural weight and students rely heavily on instructor guidance (Hofstede, 2001), perceived teacher support becomes a critical trust signal. When students lack direct BCI experience, they rely on teachers as vicarious information sources—a mechanism consistent with Bandura's (1986) social learning theory.

Behavioral intention (BI) serves as the central dependent variable in the proposed framework.

### 2.4 Formulation of hypotheses

*H1*: Performance expectancy has a significant positive effect on the intention to use BCI technology.

Performance expectancy consistently showed the strongest predictive power in existing UTAUT studies (Venkatesh et al., 2003; Dwivedi et al., 2019). In teaching contexts, students evaluate whether BCI technology can improve their learning outcomes.

*H2*: Effort expectancy has a significant positive impact on BCI technology usage intentions.

When users perceive technology as easy to learn and use, their acceptance intention increases accordingly.

*H3*: Social influence has a significant positive impact on the intention to use BCI technology.

Social influence reflects the role of significant others’ opinions on individual technology acceptance.

*H4*: Facilitating conditions have a significant positive impact on the intention to use BCI technology.

Facilitating conditions reflect the degree to which the organization and technical infrastructure support the use of technology.

*H5*: Personal innovativeness has a significant positive impact on the intention to use BCI technology.

Personal innovativeness captures an individual’s willingness to embrace new technologies.

*H6*: Neuroethical concerns are expected to negatively influence users’ intention to adopt BCI technology.

Unlike conventional educational tools, BCI interfaces directly with the brain, raising distinct ethical worries.

*H7*: Teacher support has a significant positive effect on students’ intention to use BCI technology.

Teacher support acknowledges that educators are key to promoting the use of educational technology.

## 3 Methodology

### 3.1 Research design

We used a convergent mixed methods design (Creswell and Plano Clark, 2017), running the quantitative survey and the qualitative in-depth interviews at the same time to build a more integrated picture of why people adopt BCI technology. The quantitative side tested the hypotheses in our extended UTAUT model, while the qualitative side took a root-cause approach.

### 3.2 Research objects and sampling procedure

The quantitative sample consisted of 800 students recruited from 10 universities in three provinces of China (Shandong, Jiangxi, and Chongqing) through a multi-stage stratified random sampling procedure. First, we purposively selected 10 higher education institutions representing a mix of comprehensive universities, technical colleges, and arts-focused institutions across eastern, central, and western China to ensure geographic and institutional diversity. Second, within each institution, we randomly selected two to three academic departments using a random number generator. Third, within each selected department, we recruited participants from intact classes using systematic random sampling (every fifth student on the class roster). The inclusion criteria were: (a) enrolled as a full-time student, (b) aged 18 years or above, and (c) no prior experience with BCI technology.

Of these participants, 210 (26.25%) were junior college students, while 590 (73.75%) were undergraduates. Grade distribution: 466 (58.25%) freshmen, 97 (12.13%) sophomores, 235 (29.38%) juniors, and 2 seniors (0.25%). In terms of household registration, 661 participants (82.63%) came from the eastern region, 94 (11.75%) from the central region, and 43 (5.38%) from the western region. Disciplinary backgrounds were divided into four groups: STEM (246, 30.75%), economics, management and humanities (280, 35.00%), arts (249, 31.13%), and other fields (25, 3.13%).

Table 1 summarizes the demographic characteristics of the sample. We note that the achieved sample is skewed toward first-year students (58.3%) and students from eastern China (82.6%), and that fourth-year students are underrepresented (*n* = 2), because most seniors were engaged in off-campus internships and thesis work during the data-collection period and were therefore rarely present in the intact classes that formed the sampling frame. The findings are thus most directly generalizable to early-year students in eastern Chinese higher-education institutions. Subgroup robustness analyses addressing this compositional skew are reported in Section 4.3, and this boundary is revisited in the Limitations.

**Table 1:** Demographic characteristics of the study sample (*N* = 800).

Feature variable	Category	*n* (%)
Educational level	Junior college	210 (26.3)
	Undergraduate	590 (73.7)
Year of study	First year	466 (58.3)
	Second year	97 (12.1)
	Third year	235 (29.4)
	Fourth year	2 (0.3)
Household registration	Eastern region	661 (82.6)
	Central region	94 (11.8)
	Western region	43 (5.4)

All participants provided formal written informed consent prior to data collection. The study was approved by the Academic Ethics Committee of Yantai Institute of Science and Technology (Approval No. 2024-100901) and was carried out in accordance with the ethical principles of the Declaration of Helsinki. Participants were informed that their involvement was voluntary, that they could withdraw at any time without penalty, and that all data would be anonymized and stored securely.

### 3.3 Measuring tools

The questionnaire design was based on the validated UTAUT scale and was localized and revised based on the technical characteristics of BCI. All constructs were measured on a 5-point Likert scale, ranging from 1 (strongly disagree) to 5 (strongly agree). The final instrument comprised Performance Expectancy (5 items), Effort Expectancy (4 items), Social Influence (4 items), Facilitating Conditions (5 items), Personal Innovativeness (3 items), Neuroethical Concerns (6 items), Teacher Support (3 items), and Behavioral Intention (4 items); the full item wording is provided in Appendix A. The development and validation of the two newly developed scales is detailed in Section 3.3.1.

#### 3.3.1 Development and content validity of the neuroethical concerns and teacher support scales

The two extended-UTAUT constructs were adapted from established literature rather than developed *de novo*. The Neuroethical Concerns scale (6 items) operationalizes the four ethical domains identified in the systematic scoping review of BCI ethics by Burwell et al. (2017)—privacy and data security, autonomy and cognitive liberty, personal identity, and informed consent—supplemented by related neuroethics scholarship (Ienca and Haselager, 2016; Glannon, 2014); each domain was operationalized into one to two Likert items by the research team, covering collection and storage of neural data (NC1), mental privacy (NC2), autonomy and perceived control (NC3), personal identity (NC4), unauthorized access to neural data (NC5), and equity of access (NC6). The Teacher Support scale (3 items) was adapted from widely used instructor-support measures in educational technology adoption research (Habibi et al., 2023; Farooq et al., 2017), with wording modified to reference BCI technology specifically and the Chinese educational context; the three items reflect instructor encouragement, practical assistance, and credibility as an information source.

Prior to the main data collection, the adapted items were pilot-tested with 10 students (excluded from the main sample) for comprehension, and two items were reworded for clarity based on their feedback. We note transparently that neither scale underwent formal psychometric scale development (e.g., expert panel review with content validity indexing), because the primary objective of the study was theory testing, with scale adaptation conducted to operationalize the theoretical constructs. In the present sample, both scales nonetheless demonstrated strong psychometric properties (Section 4.1): Neuroethical Concerns (Cronbach’s *α* = 0.94, CR = 0.95, AVE = 0.78, standardized loadings 0.85–0.89) and Teacher Support (α = 0.92, CR = 0.95, AVE = 0.87, loadings 0.92–0.94). The absence of full psychometric validation is acknowledged as a limitation in Section 6, where independent validation of both scales is identified as a necessary future direction. All item wording is provided in Appendix A.

### 3.4 Qualitative data gathering

After the quantitative data collection, a subsample of 40 participants was selected for semi-structured in-depth interviews using a maximum variation sampling strategy, ensuring diversity in educational level, discipline, geographic origin, and BCI attitude scores. The interview revolves around five main themes: (1) initial response to BCI technology; (2) perceived benefits and risks; (3) obstacles in the implementation process; (4) factors that promote technology acceptance; (5) suggestions for colleges and universities to promote BCI technology.

### 3.5 Data analysis methods

#### 3.5.1 Quantitative data analysis

The partial least squares structural equation model (PLS-SEM) was used for analysis via SmartPLS 4.0. The analysis process is divided into two stages: measurement model evaluation and structural model evaluation. In addition, an independent sample *t*-test and a one-way analysis of variance were used.

PLS-SEM was selected over covariance-based SEM (CB-SEM) for three reasons. First, the study is prediction-oriented and extends UTAUT with two newly developed constructs, a theory-development context for which PLS-SEM is recommended (Hair et al., 2019). Second, the data departed significantly from multivariate normality (Mardia’s multivariate skewness and kurtosis tests, both *p* < 0.001; Mardia, 1970), and PLS-SEM imposes no distributional assumptions, whereas the default maximum-likelihood estimation of CB-SEM assumes multivariate normality. Third, the model is relatively complex (eight constructs, 34 indicators), a setting in which PLS-SEM yields stable estimates. The sample size (*N* = 800) would also support CB-SEM but was not the deciding consideration. As an estimator-robustness check, the structural model was re-estimated using OLS multiple regression on construct scores (Appendix B); the pattern of significant paths and the dominance of teacher support were fully replicated (R^2^ = 0.62, versus 0.63 in PLS-SEM).

#### 3.5.2 Qualitative data analysis

Thematic analysis was employed following the six-phase framework proposed by Braun and Clarke (2006). Two researchers independently coded all 40 transcripts using an initial codebook derived from the interview protocol, refining it iteratively as new codes emerged; inter-coder agreement was high (Cohen’s *κ* = 0.87), and discrepancies were resolved through consensus discussion. Thematic saturation was assessed post-hoc by reviewing the emergence of themes across the interview series: all four themes recurred throughout the interviews, and the final interviews yielded no new themes or codes, indicating that saturation had been reached. Theme prevalence and representative quotations are reported in Section 4.4 and Table 2.

**Table 2:** Summary of qualitative themes, prevalence, and representative quotations.

Theme	Definition	Participants endorsing (*n*/40)	Quotations presented (Section 4.4)
Privacy Anxiety	Concerns about the collection, storage, access, ownership, and potential misuse of neural data	28	6 (including one contrasting case)
Technological Unfamiliarity	Skepticism about the reliability, effectiveness, and scientific maturity of BCI	22	6 (including one contrasting case)
Insufficient Institutional Readiness	Perceived lack of infrastructure, funding, technical support, and participatory decision-making at universities	25	5
Absence of Ethical Review	Perceived lack of dedicated ethics governance and clear usage boundaries for BCI deployment in education	19	5

## 4 Findings

### 4.1 Measurement model evaluation

The evaluation results of the measurement model show that each scale has good reliability and validity (Table 3). During revision, the reverse-worded personal innovativeness item PI4 was found to perform poorly (standardized loading 0.24; corrected item-total correlation 0.15) and was removed from the scale, raising the PI scale’s Cronbach’s *α* from 0.74 to 0.90; all reported results therefore use the final three-item PI scale. Cronbach’s α values ranged from 0.90 to 0.96 and all standardized loadings exceeded 0.85, confirming internal consistency. Composite reliability (CR) ranged from 0.937 to 0.967, above the recommended 0.70 threshold, and the Average Variance Extracted (AVE) ranged from 0.776 to 0.865, above the 0.50 criterion, indicating good convergent validity.

**Table 3:** Results of reliability and validity assessments (final measurement model with the three-item PI scale).

Construct	No. of items	Cronbach’s α	CR	AVE
Performance Expectancy	5	0.958	0.967	0.855
Effort Expectancy	4	0.933	0.952	0.833
Social Influence	4	0.939	0.957	0.847
Facilitating Conditions	5	0.952	0.963	0.840
Personal Innovativeness	3	0.898	0.937	0.831
Neuroethical Concerns	6	0.942	0.954	0.776
Teacher Support	3	0.922	0.950	0.865
Behavioral Intention	4	0.934	0.953	0.835

Discriminant validity was assessed with the Fornell-Larcker criterion and the heterotrait-monotrait ratio of correlations (HTMT) (Fornell and Larcker, 1981; Henseler et al., 2015). As shown in Table 4, the square root of each construct’s AVE exceeds its correlations with all other constructs, and all HTMT values in Table 5 are below the 0.85 threshold (maximum 0.807), supporting the discriminant validity of the measurement model.

**Table 4:** Fornell-Larcker discriminant validity matrix (diagonal: square root of AVE; off-diagonal: inter-construct correlations).

Construct	PE	EE	SI	FC	PI	NC	TS	BI
PE	0.925	0.551	0.683	0.650	0.610	0.231	0.592	0.588
EE	0.551	0.913	0.748	0.680	0.562	0.282	0.567	0.619
SI	0.683	0.748	0.920	0.764	0.707	0.282	0.666	0.686
FC	0.650	0.680	0.764	0.917	0.643	0.206	0.687	0.653
PI	0.610	0.562	0.707	0.643	0.912	0.294	0.651	0.655
NC	0.231	0.282	0.282	0.206	0.294	0.881	0.315	0.237
TS	0.592	0.567	0.666	0.687	0.651	0.315	0.930	0.708
BI	0.588	0.619	0.686	0.653	0.655	0.237	0.708	0.914

**Table 5:** HTMT discriminant validity matrix (all values below the 0.85 threshold).

Construct	PE	EE	SI	FC	PI	NC	TS	BI
PE	—							
EE	0.584	—						
SI	0.721	0.799	—					
FC	0.681	0.721	0.807	—				
PI	0.659	0.614	0.770	0.696	—			
NC	0.244	0.302	0.300	0.218	0.320	—		
TS	0.630	0.612	0.716	0.733	0.716	0.339	—	
BI	0.622	0.663	0.732	0.693	0.715	0.253	0.763	—

Common method bias. Because all quantitative data were self-reported via a single instrument administered at one time point, common method bias was assessed in several ways (Podsakoff et al., 2003). Procedurally, participation was anonymous and voluntary, and the instructions emphasized that there were no right or wrong answers. Statistically, Harman’s single-factor test across the 34 retained items (following the deletion of the poorly performing item PI4 described above) showed that the first unrotated factor accounted for 50.7% of the total variance—marginally above the conventional 50% heuristic—with six factors exceeding an eigenvalue of 1. We report this result frankly rather than dismissing it; however, two further results indicate that common method variance is unlikely to systematically account for the observed structural relationships. First, full collinearity variance inflation factors (VIFs) ranged from 1.16 to 4.00, all below the threshold of 5 (Kock, 2015); the highest value (4.00 for social influence) slightly exceeds the stricter 3.3 criterion, which we note transparently. Second, the inter-construct correlation pattern is strongly differentiated (e.g., *r* = 0.24 between neuroethical concerns and behavioral intention, versus *r* = 0.69 between social influence and behavioral intention), and three of the seven structural paths are non-significant—atypical of method-driven covariance, which tends to inflate correlations and significance uniformly. Taken together, while some common method variance may be present, it is unlikely to account for the complex, differentiated structural relationships reported below; single-source, single-wave self-report is nevertheless acknowledged as a limitation (Section 6). The comparatively higher VIF for social influence (4.00) reflects its expected overlap with facilitating conditions and effort expectancy at the pre-adoption stage; since all HTMT ratios remain below 0.85 and this path is small and interpreted cautiously, this level of collinearity does not threaten the structural estimates.

### 4.2 Structural model results

**Figure 1:**
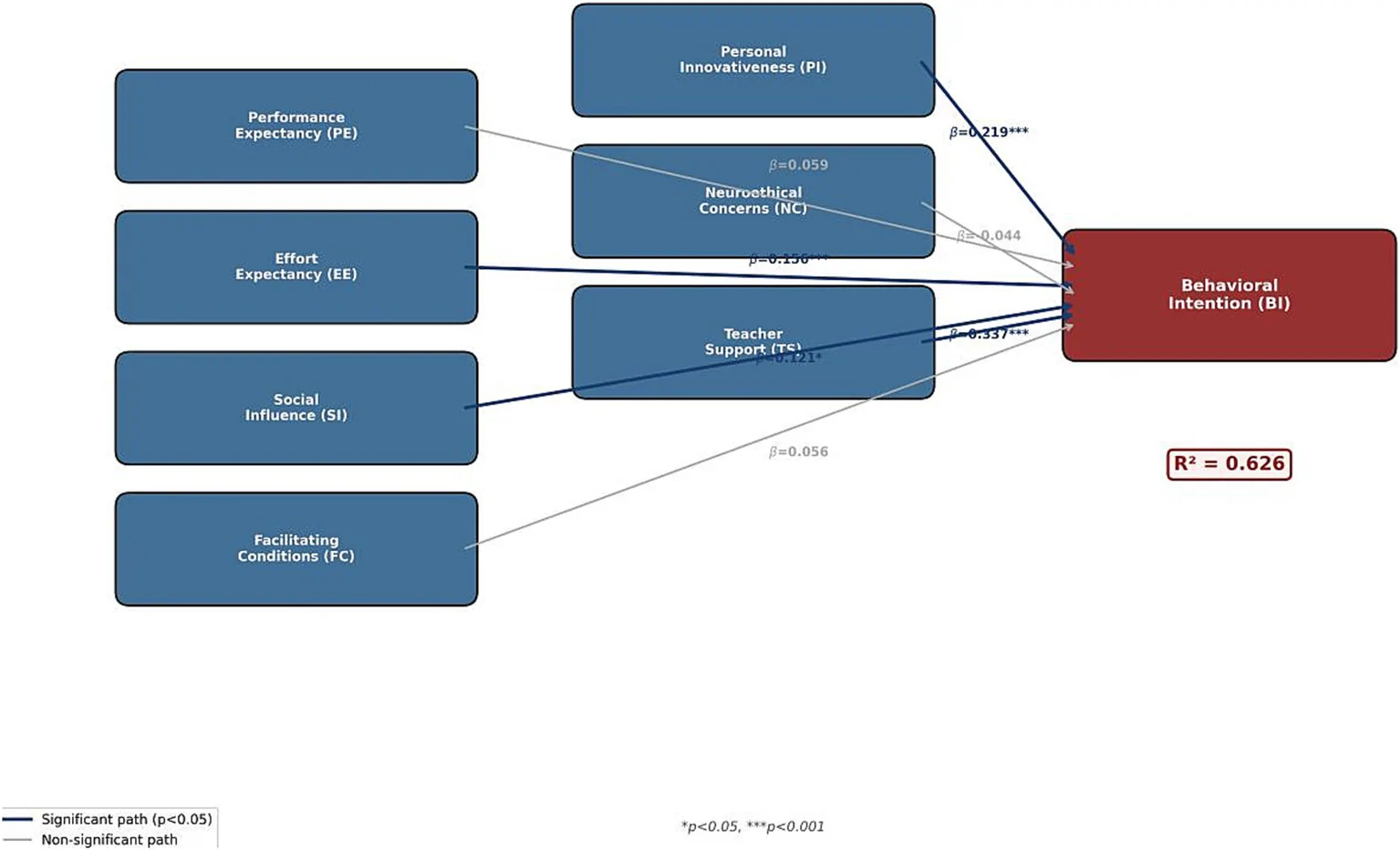
Path coefficient diagram of the extended UTAUT model (*N* = 800).

The structural model explained 62.6% of the variance in behavioral intention (R^2^ = 0.626), indicating strong explanatory power. Figure 1 shows the standardized path coefficients and their significance levels, and Table 6 reports the bootstrap test results for all hypothesized paths.

**Table 6:** Hypothesis testing results (exact two-tailed *p*-values derived from the bootstrap *t*-statistics).

Hypothesis	Path	*β*	*t*-value	*p*	Result
H1	PE → BI	0.059	1.355	0.175	Not Supported
H2	EE → BI	0.156	3.531	<0.001	Supported
H3	SI → BI	0.121	1.971	0.049	Supported
H4	FC → BI	0.056	1.073	0.283	Not Supported
H5	PI → BI	0.219	5.011	<0.001	Supported
H6	NC → BI	−0.044	1.196	0.232	Not Supported
H7	TS → BI	0.337	6.445	<0.001	Supported

The social influence path (*β* = 0.121, *t* = 1.971, *p* = 0.049) had a 95% confidence interval of [0.001, 0.241].

Effect size (f^2^) analysis showed that teacher support had a moderate effect size (f^2^ = 0.19), personal innovativeness (f^2^ = 0.12) and effort expectancy (f^2^ = 0.06) were small to medium effect sizes, and social influence (f^2^ = 0.04) was a small effect size.

This outcome pattern is significantly different from the classical UTAUT study. In Venkatesh et al. (2003), performance expectancy dominated with a path coefficient of 0.53, while in this study, the path coefficient of performance expectancy was only 0.059, which failed to reach statistical significance. This fundamental change suggests that the acceptance logic of BCI technology may be fundamentally different from that of traditional information technology.

### 4.3 Analysis of group differences

**Figure 2:**
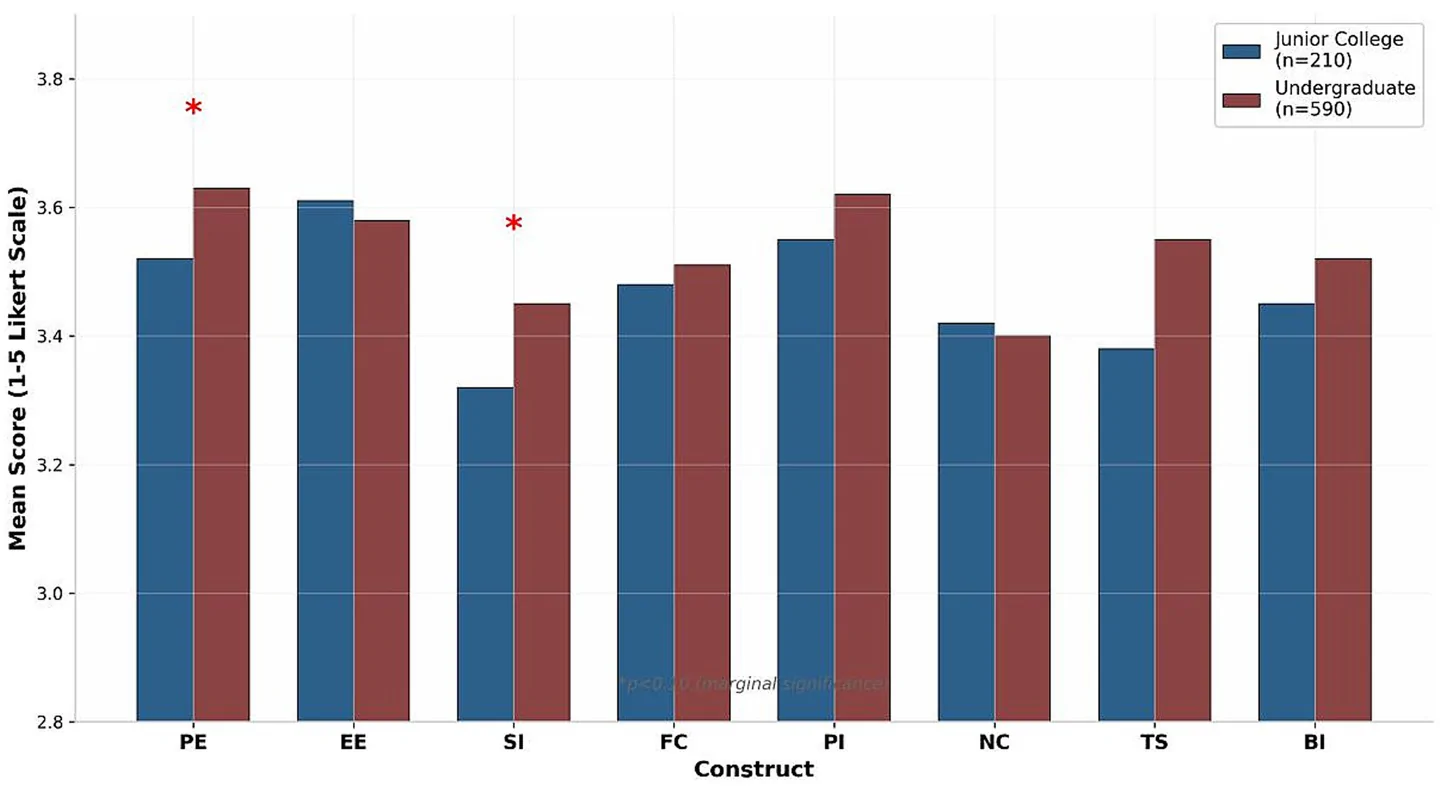
Comparison of BCI acceptance among different groups (Junior College vs. Undergraduate). *p < 0.10 (marginally significant, independent-samples t-test).

The results of the independent sample *t*-test revealed the differences between junior college students and undergraduates across the constructs. In terms of performance expectancy, undergraduate students (*M* = 3.63, SD = 0.87) scored slightly higher than junior college students (*M* = 3.52, SD = 0.84), but the difference was only significant at the marginal level (*t* = −1.671, *p* = 0.095). On the social influence side, undergraduates scored higher (*M* = 3.45, SD = 0.85) than junior college students (*M* = 3.32, SD = 0.82), approaching significance (*t* = −1.862, *p* = 0.063). Beyond that, the two groups looked largely alike. Figure 2 illustrates these group comparisons.

We also checked whether students’ disciplinary backgrounds made any difference. They did not. Across all eight constructs, not a single comparison reached significance (all *p* > 0.10), which suggests that a student’s field of study does not meaningfully shape how they view BCI.

Because the sample over-represents first-year and eastern-region students, we additionally re-estimated the model separately for eastern (*n* = 661) versus central/western students (*n* = 139) and for first-year (*n* = 466) versus non-first-year students (*n* = 334) (Appendix B). Teacher support was the strongest significant predictor in every subgroup (*β* = 0.25–0.47, all *p* < 0.001), and personal innovativeness remained significant in all four subgroups, whereas the magnitude of the effort expectancy and social influence effects varied across subgroups. Including educational level, region, and year of study as control variables in the full-sample model did not change the pattern, and none of the three controls was significant (all *p* > 0.39). The core conclusion—that teacher support dominates BCI acceptance intention at the pre-adoption stage—thus appears robust to the demographic composition of the sample, although the varying strength of secondary predictors warrants the caution expressed in the Limitations.

### 4.4 Qualitative analysis results

When we analyzed the 40 interview transcripts thematically, four barriers to implementation kept surfacing: privacy anxiety, technological unfamiliarity, insufficient institutional readiness, and the absence of ethical review.

#### 4.4.1 Privacy anxiety

According to 28 interviewees, privacy anxiety was the most common concern. A full spectrum of concerns pertaining to data collection, storage, access, and potential misuse are covered under this theme.

“I do not like the idea of my EEG data just sitting in some database. Who knows what it might be used for later—could an insurance company get hold of it? When I start looking for a job, would employers be able to look up my ‘substandard attention’ records?” (Second-year junior college, STEM, eastern region).

“The thought of a machine reading my mind honestly makes me uncomfortable, even if all it’s doing is tracking attention. This is completely different from my phone collecting my browsing history—my brain is my last private territory.” (Third-year undergraduate, economics and management, central region).

“I worry about my brain data being hacked or leaked. Once it is out, you cannot take it back. You can change a password and re-enroll a fingerprint, but can you change your brainwave pattern?” (Fourth-year undergraduate, other disciplines, eastern region).

“What scares me most is not the technology itself, but who the data belong to—me, the university, or the device manufacturer? If the university uses my EEG data in research publications, should not it ask for my consent first?” (Second-year undergraduate, economics and management, eastern region).

“They say the data are anonymized, but EEG data can identify who I am, cannot they? I read in a popular-science article that everyone’s brainwaves are unique—then is not this so-called ‘anonymity’ just self-deception?” (Third-year undergraduate, STEM, central region).

Notably, not all interviewees saw privacy as an insurmountable barrier. One fourth-year STEM undergraduate took a more permissive view: “If the university could offer a clear data-use agreement and let me view and delete my own data at any time, I would be willing to try. Privacy concerns should not be a reason to refuse, but a reason to push for better regulation.” This contrasting case suggests that institutionalized privacy safeguards may effectively ease students’ ethical concerns, downgrading privacy anxiety from a veto factor to a manageable one.

#### 4.4.2 Technological unfamiliarity

Technological unfamiliarity—voiced by 22 interviewees—primarily takes the form of skepticism over the reliability, effectiveness, and scientific foundations of BCI technology, reflecting a credibility deficit of consumer-grade EEG devices in public perception.

“Can a headband actually read my mind? It sounds like science fiction. I cannot even get noise cancellation to work properly on my earphones—why should I believe it can read my brain?” (Second-year undergraduate, arts, eastern region).

“I need to see scientific proof that this thing actually works, and that everyone is using it, before I’m willing to give it a try. I do not want to be one of the first guinea pigs.” (Third-year undergraduate, economics and management, central region).

“I have seen those consumer EEG devices online that claim to measure concentration and relaxation, but I doubt they are really accurate. If the device itself is inaccurate, aren’t all data-driven teaching decisions built on sand?” (Second-year junior college, STEM, eastern region).

“I once tried a smart wristband for sleep tracking, and the data were a mess—completely at odds with how I actually felt. Would BCI be the same? It might report that I was not paying attention when I was actually just thinking too deeply about a problem.” (Second-year junior college, STEM, western region).

“If this were used in class and my teacher saw my attention data drop, would they think I have a bad attitude toward learning? What if the device misjudges me?” (First-year undergraduate, economics and management, eastern region).

By contrast, one third-year STEM undergraduate expressed a more open stance: “Every new technology faces doubt when it first appears, but BCI is based on objective measurement of EEG signals, not mysticism. The key is transparency—if the working principle, measurement accuracy, and error margins are explained clearly, people will naturally come to trust it.” This stance suggests that the remedy for technological unfamiliarity lies in the depth and transparency of science communication.

#### 4.4.3 Insufficient institutional readiness

Insufficient institutional readiness was mentioned by 25 interviewees as a structural barrier: they perceived that universities currently lack the infrastructure, funding, and professional personnel needed to advance BCI technology.

"We haven't even built a basic AI classroom at our university, let alone BCI technology. It's not ready at all—some classrooms don't even have working projectors." (Third-year undergraduate, economics and management, central region).

"Who will provide technical support if something goes wrong? Does our IT department even understand it? It takes them ages just to fix the campus Wi-Fi." (First-year junior college, arts, eastern region).

“Rolling out this technology costs money—where would it come from? A tuition increase? Our university raised tuition just last year; another rise would be more than my family could afford.” (First-year undergraduate, STEM, western region).

“I would need a lot of training before I felt comfortable using this kind of technology. Does the university have any plan for that? Who would pay for the training? Would it end up coming out of us students’ own pockets again?” (Second-year junior college, arts, eastern region).

“I do not think the university has even started the most basic discussion—no one has asked us students whether we are willing or what we are worried about. Technology plans are pushed through top-down, and we just accept them passively.” (Third-year undergraduate, economics and management, eastern region).

At its core, insufficient institutional readiness reflects a systemic absence: hardware, technical support, funding, and participatory decision-making are all lacking. Interviewees widely perceived that universities tend to value acquisition over supporting infrastructure when introducing emerging technologies, with little integrated planning of the implementation ecosystem.

#### 4.4.4 Absence of ethical review

The absence of ethical review is another important finding: 19 interviewees stated that universities currently lack a dedicated ethical review mechanism and clear usage boundaries for BCI technology.

"We need a dedicated neuroethics committee to evaluate the use of this technique. The existing ethics review committee may not understand neurotechnology—the most complex proposal it has reviewed is probably a questionnaire survey." (First-year undergraduate, other disciplines, eastern region).

"I'm worried about fairness. Can all students use it equally, or can only those with good economic conditions enjoy it? If BCI really does improve learning efficiency, wouldn't students who cannot afford it be losing from the very start?" (Third-year undergraduate, economics and management, western region).

“Who decides where the boundaries of BCI use in education lie? There need to be clear usage guidelines. For example, could BCI data be used to evaluate teachers’ teaching, or to screen for ‘problem students’?” (Third-year undergraduate, economics and management, central region).

“If a student does not want to take part, can they withdraw at any time without penalty? These things have to be settled before implementation. I do not want to be labeled ‘uncooperative’ by my teachers just because I refused.” (Second-year junior college, arts, eastern region).

“If the data show that a student has attention problems, will teachers single them out? Will it affect scholarships and awards? Will these data end up in my academic record?” (Second-year junior college, arts, eastern region).

The core tension behind the absence of ethical review lies in the lag of capacity and the vacuum of authority: the existing academic ethics review system is ill-equipped—in membership, expertise, and procedure—for the particularities of neurotechnology, while fundamental questions about who should make the rules, and on what principles, remain unanswered. This double absence leaves students with neither institutional protection nor a channel for participation when facing BCI technology.

Taken together, privacy anxiety and insufficient institutional readiness were the most widely shared concerns (mentioned by 28 and 25 interviewees, respectively), followed by technological unfamiliarity (22 interviewees) and the absence of ethical review (19 interviewees). The focus of concern varied across student backgrounds: STEM students were more inclined to question the scientific basis of the technology, students in economics, management, and the humanities focused more on institutional design and fairness, and arts students were more sensitive to privacy and identity issues. These differences suggest that universities should adopt differentiated communication and education strategies when introducing BCI technology, rather than a one-size-fits-all rollout.

## 5 Discussion

### 5.1 Interpretation of key findings

What we saw in the quantitative data did not line up well with what traditional technology acceptance models would lead you to expect. In the original UTAUT framework (Venkatesh et al., 2003), performance expectancy had a path coefficient of 0.53 and was clearly the strongest predictor—a finding later confirmed by the meta-analysis of Tamilmani et al. (2021). But our results told a different story: performance expectancy came through with a coefficient of just 0.059 and did not come close to statistical significance.

We identify four interrelated mechanisms that explain why performance expectancy, effort expectancy, and social influence showed markedly weaker effects in the BCI context than in traditional technology adoption studies.

1 Cognitive abstraction barrier: BCI technology remains conceptually distant from students’ daily educational experiences. Unlike a learning management system or AI chatbot, where the performance benefit (better grades, faster completion) is immediately tangible, BCI’s educational value is mediated through complex neurophysiological mechanisms that students cannot easily visualize. Trope and Liberman's (2010) construal level theory suggests that when benefits are psychologically distant, they fail to activate strong behavioral intentions.
2 Technology immaturity: BCI for education is still largely at the experimental stage. Without established success stories, testimonials, or visible peers benefiting from the technology, students have no concrete referents upon which to build performance expectations. This differs fundamentally from studying mature technologies where widespread adoption provides social proof.
3 Ethical overshadowing: The salience of neuroethical concerns may cognitively overshadow performance considerations. When students encounter a technology that reads their brain activity, ethical worries (privacy, autonomy, identity) become the primary focus, pushing utilitarian calculations to the background. Burwell et al. (2017) noted that ethical concerns in BCI are qualitatively different from privacy concerns in conventional technologies because they touch upon the core of personal identity.
4 Digital-native ease normalization: Contemporary college students, as digital natives, have normalized the effort of learning new interfaces. Thus, effort expectancy no longer discriminates between adoption intentions as it did in early technology acceptance studies. However, this normalization effect does not extend to trust considerations, which is why teacher support emerges as the dominant predictor.

We also interpret the social-influence path cautiously: it reached only marginal significance (*β* = 0.121, *p* = 0.049) with a small effect size (f^2^ = 0.04), although an OLS re-estimation corroborated its direction and magnitude (*β* = 0.13, 95% CI [0.04, 0.21], *p* = 0.003; Appendix B). We therefore characterize social influence as a weak, directionally consistent secondary predictor rather than a determinant on par with teacher support or personal innovativeness.

Viewed through the lens of technology acceptance theory, the study revealed that teacher support, rather than performance expectancy, is the strongest predictor (*β* = 0.337). This contradicts the basic notions of the UTAUT model and unveils that BCI technology acceptance follows a distinct psychological mechanism. Bandura’s (1986) concept of vicarious experience can explain this phenomenon: when students lack direct usage experience, they rely on teachers as a credible information source. This pattern is consistent with a trust-based acceptance mechanism in which teacher support, rather than performance expectancy, is the dominant correlate of intention at the pre-adoption stage; given the cross-sectional design, this interpretation is associational and does not establish causality.

Personal innovativeness (*β* = 0.219) has a significant effect, supporting the hypothesis of Agarwal and Prasad (1998). For emerging technologies such as BCI, highly innovative individuals are more likely to be early adopters, subsequently fueling the spread through peer influence and social learning mechanisms.

### 5.2 Comparison with previous studies

**Figure 3:**
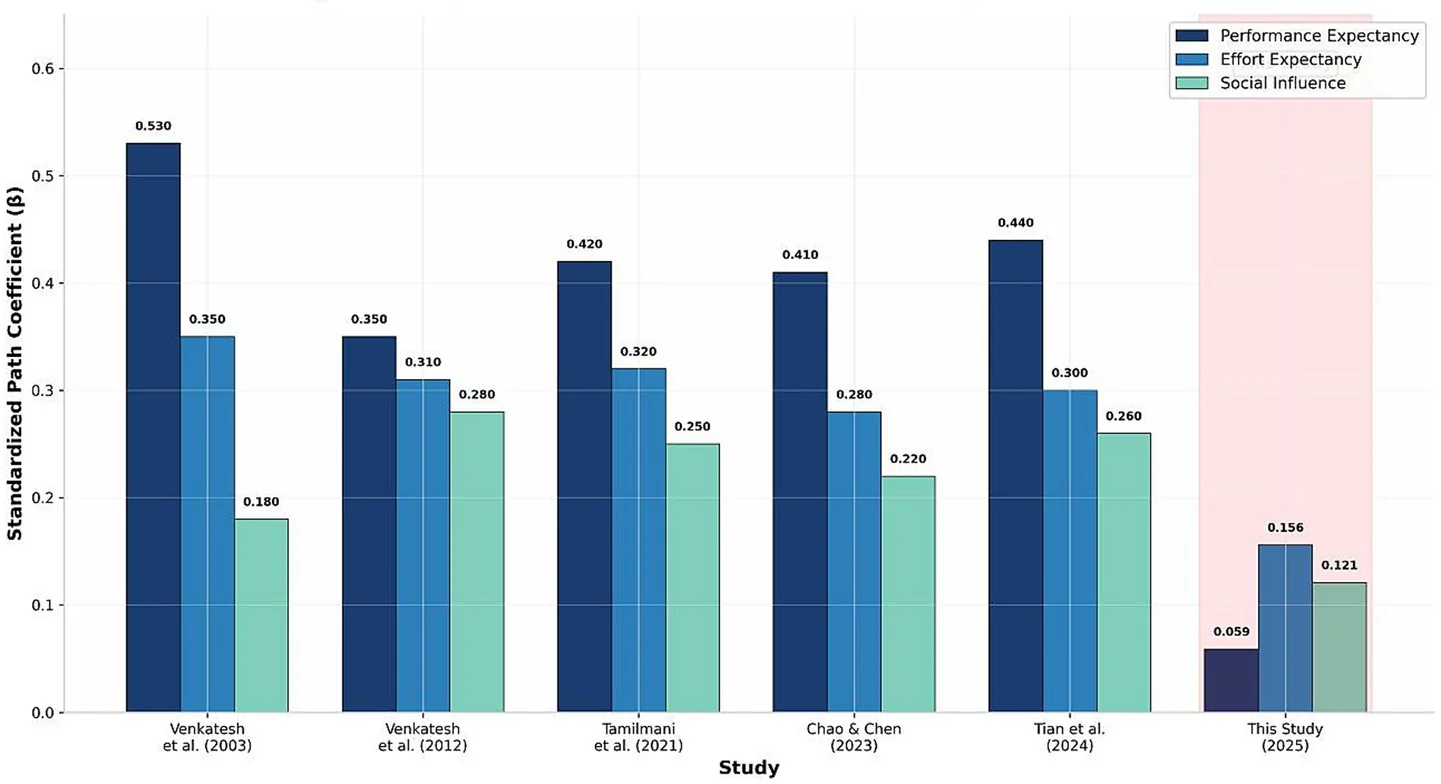
Comparison of UTAUT path coefficients across representative studies.

This paper presents a systematic comparison of representative UTAUT studies from around the world (Figure 3). The coefficient of performance expectancy in this study was only 0.059, much lower than all previous studies. This contrasts with the 0.44 found by Tian et al. (2024) and the 0.41 reported by Chao and Chen (2023), suggesting that BCI technology is fundamentally distinct from traditional educational techniques in terms of acceptance logic. The coefficient of effort expectancy was 0.156, also lower than in previous studies, reflecting the increasing flexibility of the current user group. The coefficient of social influence was 0.121, still significant but lower than classic studies (0.18–0.35), possibly due to the highly personal nature of BCI technology.

### 5.3 Neuroethical analysis

The quantitative and qualitative strands appear, at first sight, to diverge: neuroethical concerns did not significantly predict intention (*β* = −0.044, *p* > 0.05), whereas ethical unease was pervasive in the interviews. Three considerations reconcile this pattern. First, concerns are widespread but moderate rather than prohibitive: 87.4% of respondents scored at or above the NC scale midpoint and 29.6% averaged “agree” or higher, indicating salient rather than rejecting concern. Second, the zero-order association between NC and intention is in fact positive (*r* = 0.24, *p* < 0.001)—students who are more aware of and engaged with BCI report both higher concern and higher intention—and the small negative path coefficient emerges only after teacher support, personal innovativeness, and social influence are partialled out. Neuroethical concern thus behaves as a correlate of engagement rather than a deterrent at the pre-adoption stage; notably, the negative partial association was concentrated among first-year students (Appendix B), suggesting that concerns weigh more heavily for students with the least campus experience. Third, the two instruments capture different facets of the phenomenon: survey items measure abstract worry about a technology participants had never used, whereas interviews elicited concrete, situated concerns (data ownership, secondary use, equitable access) that shape how students expect deployment to be governed rather than whether they intend to use it (Friedrich et al., 2021). Table 7 integrates the two strands by concern domain.

**Table 7:** Joint display integrating quantitative and qualitative evidence on neuroethical concerns.

Concern domain (items)	Quantitative evidence	Qualitative evidence	Integrated interpretation
Neural-data privacy (NC1, NC5)	*M* = 3.42; 42.6–42.9% agreement; NC–BI zero-order *r* = 0.24	Privacy anxiety (28/40 interviews): “You can change a password and re-enroll a fingerprint, but can you change your brainwave pattern?”	Concerns are prevalent but coexist with intention; they shape governance expectations rather than deter use
Mental privacy & autonomy (NC2, NC3)	*M* = 3.38–3.45; 40.6–44.0% agreement	“My brain is my last private territory.”	Abstract worry is salient yet does not suppress pre-adoption intention (partial *β* = −0.044, ns)
Identity (NC4)	*M* = 3.36; 39.2% agreement	“Everyone’s brainwaves are unique—then is not this so-called anonymity just self-deception?”	Identity concerns emerge mainly in interviews, where concrete scenarios are discussed
Equity of access (NC6)	*M* = 3.39; 40.6% agreement	“Can all students use it equally, or can only those with good economic conditions enjoy it?”	Fairness concerns relate to deployment governance, echoing the ethical-review theme

**Figure 4:**
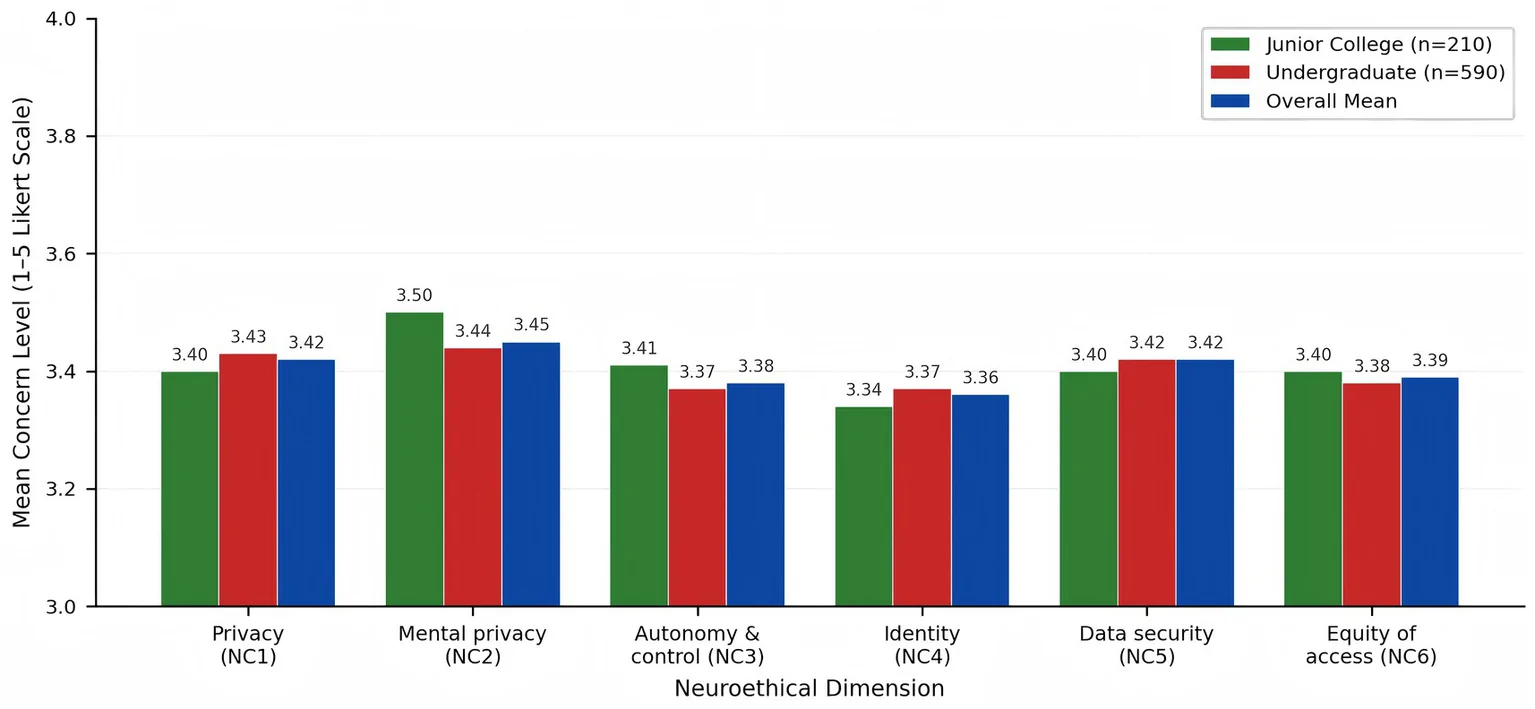
Comparison of neuroethical concerns across different groups.

Concern levels were broadly similar across domains (overall means 3.36–3.45), with neural-data privacy and mental privacy scoring highest and identity concerns lowest (Figure 4). Identity threats refer to the fear that BCI technology could fundamentally change the essence of the “self” (Ienca and Haselager, 2016). Autonomy risks remained salient, echoing Klein’s (2015) concept of ‘internal coercion’. In this regard, Lavazza and Giorgi (2023) provide a philosophical foundation for the right to mental integrity in the age of neurotechnologies, arguing that any unauthorized access to neural data constitutes a violation of the fundamental right to cognitive liberty. Informed-consent concerns likewise reveal students’ awareness of BCI technical information asymmetry (Haselager et al., 2009). Figure 4 compares the levels of neuroethical concern across demographic groups.

### 5.4 Root cause analysis

#### 5.4.1 Privacy anxiety

What’s driving privacy anxiety is the uncertainty around who controls the data and how it might be misused. Ienca and Haselager (2016) laid this out clearly: once brain data is collected, it’s vulnerable to hacking, commercial exploitation, and government surveillance.

#### 5.4.2 Technological unfamiliarity

When people struggle with a technology, it often comes down to gaps in scientific literacy and lack of hands-on experience. Kögel and Wolbring (2020) found that hearing from early BCI adopters can be a powerful way to shift attitudes.

#### 5.4.3 Insufficient institutional readiness

Lack of resources and expertise signals insufficient institutional readiness. Systematic capacity building is needed for sustainable implementation.

#### 5.4.4 Absence of ethical review

The absence of ethical review reflects the lag in moral governance of new technologies. Ligthart et al. (2023) pointed out that Chile and Spain have included neural rights in their constitutional protection frameworks.

### 5.5 Theoretical contributions

First, we integrate neuroethical concerns into the UTAUT model as an independent construct, expanding the framework’s ability to explain neurotechnology specificity. Second, the finding that teacher support, rather than performance expectancy, is the strongest correlate of intention in the BCI context challenges the default assumptions inherited from UTAUT applications to conventional IT. Third, root cause analysis introduces methodological innovation. Fourth, this research identifies unique patterns of BCI acceptance by comparing studies at home and abroad. Fifth, this study addresses the research gap regarding BCI educational applications in China (Xia and Yang, 2024).

### 5.6 Cultural context: the role of Chinese educational norms

The findings of this study must be interpreted within the specific cultural context of Chinese higher education. Several cultural factors may have shaped the observed pattern of results. First, China scores high on Hofstede’s (2001) power distance dimension, meaning students tend to defer to authority figures—particularly teachers—when evaluating novel technologies. This cultural trait likely amplified the effect of teacher support, making it the strongest predictor of BCI acceptance intention.

Second, the collectivist orientation of Chinese culture means that social influence operates through different mechanisms than in individualist Western contexts. Peer and family opinions carry substantial weight, but our finding that social influence was relatively weak (*β* = 0.121) suggests that BCI’s deeply personal nature—interfacing directly with one’s brain—may partially override collectivist norms. Students may view BCI adoption as an intensely private decision that cannot be delegated to social consensus.

Third, the examination-oriented culture of Chinese higher education means that students are highly pragmatic about technology adoption. Technologies that demonstrably improve test scores or academic efficiency are readily embraced, while those with unclear academic benefits face skepticism. This pragmatism may partly explain why performance expectancy failed to reach significance—students could not see a clear pathway from BCI to better academic outcomes.

Finally, the rapid pace of educational technology adoption in China—fueled by government initiatives such as Education Informatization 2.0—has created a population of students who are technologically adept but also cautious about hype-driven innovations. This dual orientation—tech-savvy yet skeptical—may explain why effort expectancy was significant but modest, and why personal innovativeness played a key role in distinguishing adopters from non-adopters.

## 6 Conclusions, practical implications, and future directions

In the BCI context, teacher support (*β* = 0.337), rather than performance expectancy, was the strongest correlate of intention—a notable departure from classic UTAUT findings. Personal innovativeness (*β* = 0.219), effort expectancy (*β* = 0.156), and social influence (*β* = 0.121) also mattered. Performance expectancy and facilitating conditions did not reach significance, likely because BCI’s educational value is still unproven.

Neuroethical concerns threw up an interesting paradox: statistically non-significant (*β* = −0.044), yet powerfully present in interviews. We saw no meaningful differences across education levels or disciplines. The qualitative data pointed to four barriers: privacy anxiety, technological unfamiliarity, weak institutional readiness, and a missing ethical review process.

On the practical side, we outline a phased roadmap—from laying the groundwork through small-scale pilots to full integration. Running through this framework are six principles: informed consent, data minimization, transparency, equitable access, pedagogical alignment, and continuous assessment.

This study has several limitations. First, the cross-sectional, single-wave design cannot establish causality: all reported paths represent associations, and the directionality implied by the theoretical model requires validation with longitudinal or experimental designs (Section 6.1). Second, the sample is skewed toward first-year students (58.3%) and students from eastern China (82.6%), with fourth-year students underrepresented; although subgroup robustness analyses supported the stability of the core findings (Section 4.3), the results are most directly generalizable to early-year students in eastern Chinese higher-education institutions, and balanced multi-region, multi-year samples are needed. Third, all quantitative data were self-reported via a single instrument at one time point; procedural and statistical assessments indicated that common method bias is unlikely to account for the findings (Section 4.1), but multi-informant or objective measures would strengthen future work. Fourth, the Neuroethical Concerns and Teacher Support scales were adapted from established literature and pilot-tested for comprehension, but were not subjected to full psychometric validation (e.g., expert panel review with content validity indexing); future research should independently validate these scales. Finally, the sample was drawn from 10 universities in three regions and may not represent the full diversity of Chinese higher education institutions.

### 6.1 Directions for future research

Building on the limitations and findings of this study, we propose the following avenues for future research:

1 Longitudinal adoption studies: Future research should employ longitudinal designs to track how acceptance intentions translate into actual BCI usage over time. Such studies could capture the dynamic evolution of performance expectancy as users gain direct experience with the technology.
2 Cross-country comparisons: Given the cultural specificity of our findings—particularly the dominant role of teacher support in the high-power-distance Chinese context—comparative studies across countries with different cultural profiles (e.g., individualist vs. collectivist, high vs. low power distance) would illuminate how cultural norms shape BCI acceptance mechanisms.
3 Actual usage behavior: This study focused on behavioral intention rather than actual usage. Future research should investigate post-adoption experiences, including how initial concerns about privacy and ethics evolve with continued use, and whether actual BCI usage leads to measurable learning outcomes.
4 Disciplinary differences: While our study found no significant disciplinary differences, this may be due to the pre-experimental nature of BCI in education. As BCI applications become more discipline-specific (e.g., neuroscience labs, cognitive training in psychology, attention monitoring in language learning), disciplinary differences may emerge.
5 Neuroethical concern measurement refinement: The non-significance of neuroethical concerns in the quantitative model, contrasted with their prominence in qualitative data, suggests a need for more sensitive measurement instruments. Contextualized measurement techniques—such as scenario-based assessments or deliberative polling methods—may better capture the layered nature of neuroethical decision-making.
6 Teacher perspective studies: Since teacher support emerged as the strongest predictor, understanding teachers’ own attitudes, concerns, and training needs regarding BCI is a critical next step. Without teacher buy-in, student acceptance may not translate into institutional adoption.
7 Intervention studies: Experimental designs testing specific interventions—such as hands-on BCI workshops, neuroethics education modules, or institutional readiness programs—would provide causal evidence for strategies to enhance acceptance and address barriers.

## Data Availability

The raw data supporting the conclusions of this article will be made available by the authors, without undue reservation.
