# Supplementary material for: A study on Chinese college students’ acceptance of brain-computer interface technology and its underlying logic—an empirical analysis based on the extended UTAUT model
Source: Front Hum Neurosci. 2026 Aug 21;20:1884532. doi: 10.3389/fnhum.2026.1884532 (PMC13541772; doi:10.3389/fnhum.2026.1884532)
Supplement: Supplementary file 1 [file Table_1.docx]

Supplementary Material

*for: A Study on Chinese College Students' Acceptance of Brain-Computer Interface Technology and Its Underlying Logic— An Empirical Analysis Based on the Extended UTAUT Model*

Jing Gao, Jianfeng Hu, Yang Chai, Weiyong Ning

Frontiers in Human Neuroscience | Manuscript ID 1884532

# Appendix A. Survey Instrument (Final 34 Items, English Translation)

All items were administered in Chinese using a 5-point Likert scale (1 = strongly disagree, 5 = strongly agree); the English wording below was translated by the authors for reporting purposes. The reverse-worded personal innovativeness item PI4 was removed during revision (Section 4.1).

| **Construct** | **Item** | **Wording (English translation)** |
| --- | --- | --- |
| Performance Expectancy | PE1 | Using BCI can improve my learning efficiency. |
| Performance Expectancy | PE2 | Using BCI can enhance my academic performance. |
| Performance Expectancy | PE3 | BCI can help me achieve my learning goals effectively. |
| Performance Expectancy | PE4 | BCI can help me better manage my attention. |
| Performance Expectancy | PE5 | BCI is very helpful for my learning. |
| Effort Expectancy | EE1 | Learning to use BCI would be easy for me. |
| Effort Expectancy | EE2 | My interaction with BCI would be clear and understandable. |
| Effort Expectancy | EE3 | I could easily master how to use BCI. |
| Effort Expectancy | EE4 | The BCI interface is clear and easy to understand. |
| Social Influence | SI1 | My teachers think I should use BCI. |
| Social Influence | SI2 | My classmates think BCI is helpful for learning. |
| Social Influence | SI3 | People who are important to me support my use of BCI. |
| Social Influence | SI4 | People who are important to me think I should use BCI. |
| Facilitating Conditions | FC1 | My university provides sufficient technical support for BCI. |
| Facilitating Conditions | FC2 | My university has the resources and facilities to support BCI use. |
| Facilitating Conditions | FC3 | I could get timely help if I encountered problems using BCI. |
| Facilitating Conditions | FC4 | I have the necessary conditions to use BCI. |
| Facilitating Conditions | FC5 | BCI is compatible with other technologies I use. |
| Personal Innovativeness | PI1 | I take the initiative to try new technologies. |
| Personal Innovativeness | PI2 | I am often among the first to try new technologies. |
| Personal Innovativeness | PI3 | I like trying new technologies. |
| Neuroethical Concerns | NC1 | I am concerned about my EEG data being collected and stored. |
| Neuroethical Concerns | NC2 | I am concerned that BCI may violate my mental privacy. |
| Neuroethical Concerns | NC3 | I feel I might lose control over my own thoughts. |
| Neuroethical Concerns | NC4 | I am concerned that BCI may affect my sense of self-identity. |
| Neuroethical Concerns | NC5 | I am concerned about unauthorized access to my brain data. |
| Neuroethical Concerns | NC6 | I am concerned that BCI may create inequity in education. |
| Teacher Support | TS1 | My teachers support me in using BCI for learning. |
| Teacher Support | TS2 | My teachers would provide the necessary help for using BCI. |
| Teacher Support | TS3 | My teachers believe BCI is helpful for learning. |
| Behavioral Intention | BI1 | I intend to use BCI in the near future. |
| Behavioral Intention | BI2 | I will use BCI within the next 12 months. |
| Behavioral Intention | BI3 | I plan to use BCI for my learning. |
| Behavioral Intention | BI4 | I would recommend BCI to my classmates. |

# Appendix B. Supplementary Robustness Analyses (OLS Re-estimation of the Structural Model)

Table B1 reports standardized coefficients from OLS regressions of behavioral intention on the construct scores, for the full sample and for demographic subgroups. *p < 0.05, **p < 0.01, ***p < 0.001. In the full-sample model with educational level, region, and year of study added as control variables, all three controls were non-significant (all p > 0.39) and the pattern of structural paths was unchanged. The OLS estimates corroborate the PLS-SEM results, including the dominance of teacher support and the marginal but directionally consistent social-influence path.

**Table B1. Subgroup and Full-sample OLS Robustness Analyses**

| **Path** | **Full (n=800)** | **Eastern (n=661)** | **Central/Western (n=139)** | **First-year (n=466)** | **Non-first-year (n=334)** |
| --- | --- | --- | --- | --- | --- |
| PE → BI | 0.068* | 0.042 | 0.183* | 0.104** | -0.002 |
| EE → BI | 0.158*** | 0.140*** | 0.194* | 0.240*** | 0.081 |
| SI → BI | 0.129** | 0.164*** | -0.018 | 0.064 | 0.193* |
| FC → BI | 0.055 | 0.070 | -0.040 | -0.027 | 0.140* |
| PI → BI | 0.183*** | 0.189*** | 0.180* | 0.204*** | 0.187** |
| NC → BI | -0.033 | -0.033 | -0.059 | -0.085** | 0.044 |
| TS → BI | 0.346*** | 0.326*** | 0.464*** | 0.420*** | 0.252*** |
| R² | 0.619 | 0.611 | 0.685 | 0.673 | 0.570 |
